# Evaluating contouring accuracy and dosimetry impact of current MRI-guided adaptive radiation therapy for brain metastases: a retrospective study

**DOI:** 10.1007/s11060-024-04583-9

**Published:** 2024-02-01

**Authors:** Bin Wang, Yimei Liu, Jun Zhang, Shaohan Yin, Biaoshui Liu, Shouliang Ding, Bo Qiu, Xiaowu Deng

**Affiliations:** 1 Department of Radiation Oncology, State Key Laboratory of Oncology in South China, Guangdong Provincial Clinical Research Center for Cancer, Sun Yat-Sen University Cancer Center, 651 East Dongfeng Road, Guangzhou, Guangdong 510060 People’s Republic of China; 2 Department of Radiology, State Key Laboratory of Oncology in South China, Guangdong Provincial Clinical Research Center for Cancer, Sun Yat-Sen University Cancer Center, Guangzhou, 510060 People’s Republic of China

**Keywords:** Adaptive radiotherapy (ART), MR-guided adaptive radiotherapy (MRgART), Brain metastases, Target contouring, Fractionated stereotactic radiotherapy (FSRT), MR-Linac

## Abstract

**Background:**

Magnetic resonance imaging (MRI) guided adaptive radiotherapy (MRgART) has gained increasing attention, showing clinical advantages over conventional radiotherapy. However, there are concerns regarding online target delineation and modification accuracy. In our study, we aimed to investigate the accuracy of brain metastases (BMs) contouring and its impact on dosimetry in 1.5 T MRI-guided online adaptive fractionated stereotactic radiotherapy (FSRT).

**Methods:**

Eighteen patients with 64 BMs were retrospectively evaluated. Pre-treatment 3.0 T MRI scans (gadolinium contrast-enhanced T1w, T1c) and initial 1.5 T MR-Linac scans (non-enhanced online-T1, T2, and FLAIR) were used for gross target volume (GTV) contouring. Five radiation oncologists independently contoured GTVs on pre-treatment T1c and initial online-T1, T2, and FLAIR images. We assessed intra-observer and inter-observer variations and analysed the dosimetry impact through treatment planning based on GTVs generated by online MRI, simulating the current online adaptive radiotherapy practice.

**Results:**

The average Dice Similarity Coefficient (DSC) for inter-observer comparison were 0.79, 0.54, 0.59, and 0.64 for pre-treatment T1c, online-T1, T2, and FLAIR, respectively. Inter-observer variations were significantly smaller for the 3.0 T pre-treatment T1c than for the contrast-free online 1.5 T MR scans (*P* < 0.001). Compared to the T1c contours, the average DSC index of intra-observer contouring was 0.52‒0.55 for online MRIs. For BMs larger than 3 cm^3^, visible on all image sets, the average DSC indices were 0.69, 0.71 and 0.64 for online-T1, T2, and FLAIR, respectively, compared to the pre-treatment T1c contour. For BMs < 3 cm^3^, the average visibility rates were 22.3%, 41.3%, and 51.8% for online-T1, T2, and FLAIR, respectively. Simulated adaptive planning showed an average prescription dose coverage of 63.4‒66.9% when evaluated by ground truth planning target volumes (PTVs) generated on pre-treatment T1c, reducing it from over 99% coverage by PTVs generated on online MRIs.

**Conclusions:**

The accuracy of online target contouring was unsatisfactory for the current MRI-guided online adaptive FSRT. Small lesions had poor visibility on 1.5 T non-contrast-enhanced MR-Linac images. Contour inaccuracies caused a one-third drop in prescription dose coverage for the target volume. Future studies should explore the feasibility of contrast agent administration during daily treatment in MRI-guided online adaptive FSRT procedures.

## Introduction

Online adaptive radiotherapy (ART) has attracted increasing attention in the field of radiotherapy in recent years [1–4]. New modalities and software have been introduced for online ART in clinical practices [5–7]. Recent studies have demonstrated the dosimetric advantages of online ART over conventional radiotherapy [8–10]. However, concerns have been raised regarding the accuracy of online treatment target delineation and modification [11]. Accurate online contouring is limited by time and online image quality in the online ART workflow, especially when the image quality is considerably inferior to pre-treatment planning images. Therefore, before conducting online ART, a comprehensive study of contouring accuracy based on online images is essential to set the baseline confidence levels for online contouring.

Brain metastases (BMs) are a major complication in various malignant carcinomas, particularly among patients with advanced lung cancer. Stereotactic radiosurgery (SRS) and stereotactic radiotherapy (SRT) have emerged as primary treatment modalities due to BMs exhibiting a favourable response to radiation doses [12, 13]. Previously, radiotherapy of BMs was carried out without taking into account inter-fractional anatomical changes [14–16]. However, recent studies have revealed that inter-fractional target displacement and tumour regression are frequent occurrences in fractionated radiotherapy for BMs [17–19]. In some cases, inter-fractional GTV changes exceeded primary PTV margin and lead to significant drop in GTV dose coverage [17, 18].

Although on-board radiological images such as kV/MV planar images and cone beam computed tomography showed good usability for daily setup correction, the lack of contrast for brain mass tissues limited its use for treatment target delineation or modification. Recent developments in MR-guided radiotherapy have provided new insights into the treatment of this disease. The improved soft-tissue contrast and the potential of multi-contrast images provided possibility for treatment target modification during the treatment course. Thus, the feasibility of MR-guided Fractionated Stereotactic Radiotherapy (FSRT) has been studied at multiple medical institutions [20, 21].

Former studies mainly focused on dosimetry aspects of on-board MRI guided radiotherapy of brain metastases, few research has been done to investigate accuracy of online target delineation. A recent study has found significant smaller clinical target volume (CTV) volume compared to diagnostic T1c in online-FLAIR based contouring in MR guided FSRT of postoperative BMs [11]. Whereas, accuracy of gross target volume (GTV) delineation in online MRI guided FSRT of BMs was still unknown. Based on the previous studies and the fact that daily GBCA administration was not a common practice in current MRI guided FSRT of BMs, the accuracy of brain target delineation in online MRgART requires evaluation. Baseline contour differences based on non-enhanced MRI scans should be set, and the expected influence on prescription dose coverage (PDC) should be evaluated.

## Methods

### Patient selection

Eighteen patients with 64 BMs were selected for this retrospective study. All patients had primary lung adenocarcinoma. Among them, eight patients had a single BM, 10 patients had multiple brain lesions, and one patient had 18 visible brain lesions. This study was approved by the institutional ethics committee, and all patients provided written informed consent prior to enrolment.

### MRI scanning

Before radiotherapy, all patients underwent CT scanning (Philips Brilliance™, Netherlands) and MRI scanning (Philips Ingenia 3.0 T, Netherlands) in the treatment position. A head immobilisation mask (Klarity Co., China) and a patient-specific polyurethane foam immobilisation device (Forrad Co., China) were used for body positioning. During the MRI scan, a gadolinium-based contrast agent (GBCA) was administered to the patients, and a contrast-enhanced T1w (T1c) scan (flip angle: 12◦, TE: 2.4 ms, TR: 5.0 ms, voxel size: 0.74 × 0.74 × 2.0) was obtained 15 s later, which was clinically used as the reference image for tumor contouring.

Treatment was carried out using a 1.5 T MR-Linac (Unity, Elekta AB, Stockholm, Sweden) within one week (five workdays) of the simulation scans. During the first treatment, multiple MRI scans (T1, T2, and FLAIR) were obtained online for treatment guidance and transferred to the Monaco treatment planning system (TPS) (v.5.40.02; Elekta AB, Stockholm, Sweden) for this retrospective study. The online MRI scan protocols were as follows: T1: flip angle: 8◦, TE: 3.6 ms, TR: 8.0 ms, voxel size: 0.58 × 0.58 × 2.0; T2: flip angle: 90◦, TE: 295 ms, TR: 2100 ms, voxel size: 0.58 × 0.58 × 2.0; FLAIR: flip angle: 40◦, TE: 298 ms, TR: 4800 ms, voxel size: 0.58 × 0.58 × 2.0. All the online MRI scans were performed without GBCA injections.

### Contouring

All images were transferred to the MIM Maestro (Version 7.1.3) workstation, and contouring was performed using MIM software (MIM Software, Cleveland, USA). Five radiation oncologists participated in the contouring process. GTVs were independently contoured on pre-treatment and online MRIs with different contrasts (GTV-T1c, GTV-T1, GTV-T2 and GTV-FLAIR). To eliminate the influence of the observer's memory of previous contours when working on different contrast sequences, each observer was required to contour all images of the same contrast sequence before moving on to the MRI scans of different contrast. In clinical practice, radiological reports are referred to when contouring targets. However, in this study, radiation oncologists were not required to refer to previous diagnostic reports to evaluate the performance of each MRI scan objectively. The lesions were considered invisible if the observer failed to delineate them. However, visibility can vary among observers, indicating that lesions invisible to one observer may be delineated by another.

The contours were compared using a MIM Maestro workstation. The mean distance to agreement (MDA) and Dice Similarity Coefficient (DSC) were evaluated for inter-observer consistency (between the contours of different observers) and intra-observer consistency (between the contours of different MRI scans of each observer). To evaluate inter-observer differences, the contours of a senior radiation oncologist were chosen as the baseline for comparison. To evaluate MRI performance for different lesions, BMs were divided into two subgroups: lesions larger than 3 cm^3^ and those below 3 cm^3^ in size. For larger lesions (> 3 cm^3^) visible on all MRI scans, the similarity of the contours was evaluated between different MRI scans. For smaller lesions (< 3 cm^3^), visibility was evaluated for MRI scans with different contrasts.

### Planning

All contours were transferred to the planning CT according to the rigid registration between CT and MRI in the MIM Maestro workstation. A margin of 3 mm was applied to expand the GTVs to planning target volume (PTVs). Planning was performed in the MONACO TPS using Unity MR-Linac as the treatment device. A prescription of 30 Gy in five fractions was administered to all patients, except for one case with 18 visible lesions, who was prescribed 50 Gy for the BMs and 30 Gy for the whole brain in 10 fractions. All PTVs were required to achieve at least 95% of the PDC, with the maximum dose limited to less than 110% of the prescription dose. Doses to organs at risk (OARs), such as the eyes and brainstem, were constrained according to institutional guidelines. For each case, three treatment plans (7‒9 field steps and shoot intensity-modulated radiotherapy) were separately created, targeting PTVs generated on MR-Linac images (namely PTV-T1, PTV-T2, and PTV-FLAIR) to simulate the online adaptive planning process. PTV-T1c was used as the ground-truth target volume to evaluate the prescription dose coverage of the plans from the online MR-Linac images. The new conformity index (NCI) of the PTVs was analysed to evaluate the degree to which the prescribed isodose volume conformed to the shape and size of the target volume [22]. The NCI is defined as follows:

$${\text{NCI}}=\frac{\left(treatment\;volume\right)\;\times\;\left(prescription\;isodose\;volume\right)}{{(volume\;of\;the\;target\;covered\;by\;the\;prescription\;isodose\;volume)}^{2}}$$

### Statistical analysis

Means and standard deviations (SD) were used to describe continuous data. Statistical analysis was carried out using a paired t-test, and *P* < 0.05 was defined as the threshold for statistical significance. All analyses were performed using SPSS (v25.0) software (IBM Corporation, Armonk, NY, USA).

## Results

### Inter-observer consistency

**Fig. 1:**
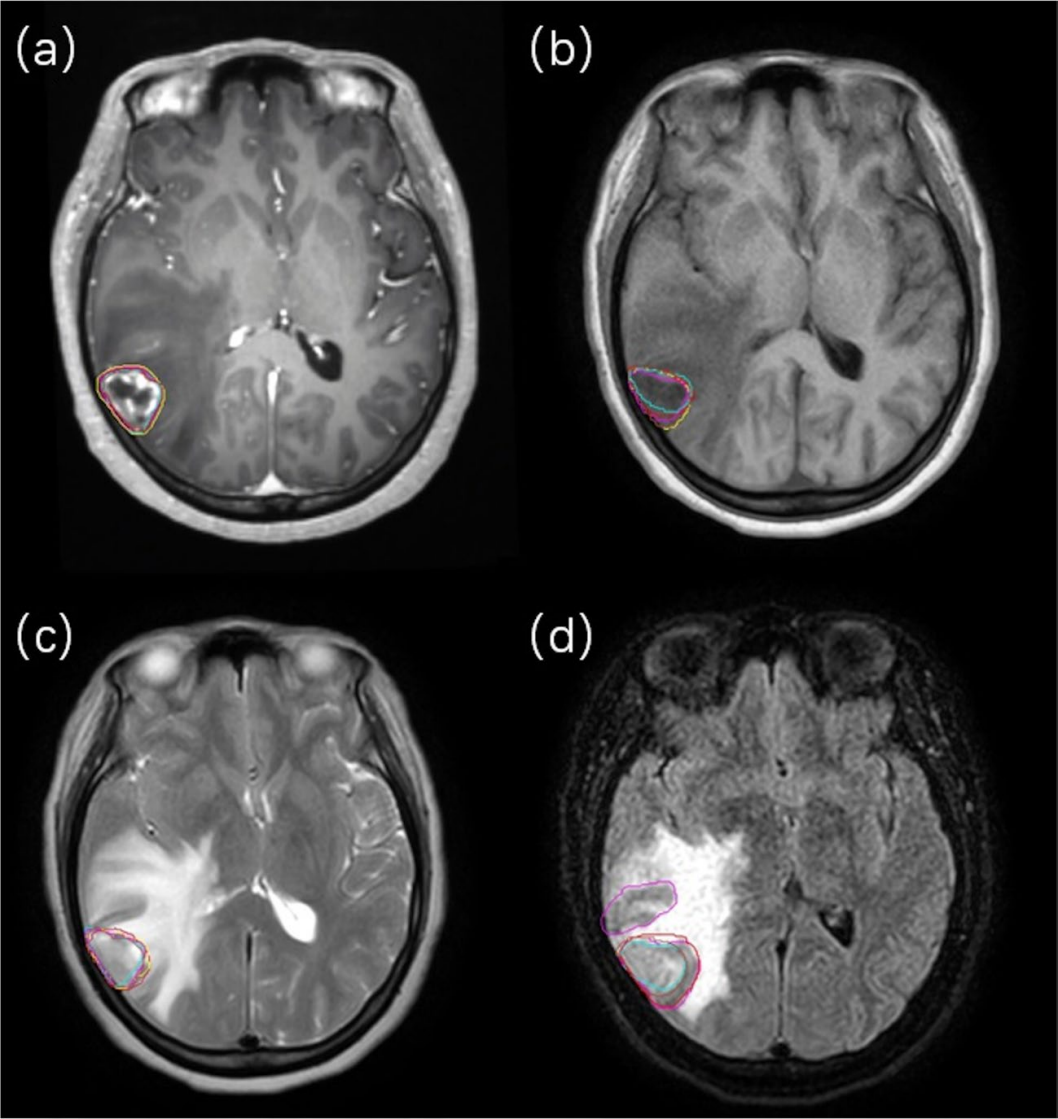
A case with a single brain metastasis (BM) larger than 3 cm^3^ as shown in different MR images (**a**) pre-treatment T1c, **b** online-T1, **c** online-T2, **d** online-FLAIR, and contours of different observers (lines with different colours)

Using the pre-treatment MR images (T1c), a senior radiation oncologist contoured 64 lesions. For the other observers, the contoured targets ranged from 50‒67. Among all lesions identified by the senior radiation oncologist, 12 were > 3 cm^3^, and 52 were < 3 cm^3^ in size. For larger lesions (≥ 3 cm^3^), pathological changes were observed in all image sets. However, different image contrasts emphasise different aspects of pathological changes, as shown in Fig. 1, which may cause confusion among observers. For smaller lesions (< 3 cm^3^), a significant reduction in the visibility of the lesions was observed in the online MR-Linac images. The average visible targets for all the observers were 59, 23.6, 32.8, and 36.8 for the pre-treatment T1c, online-T1, T2, and FLAIR, respectively.

**Fig. 2:**
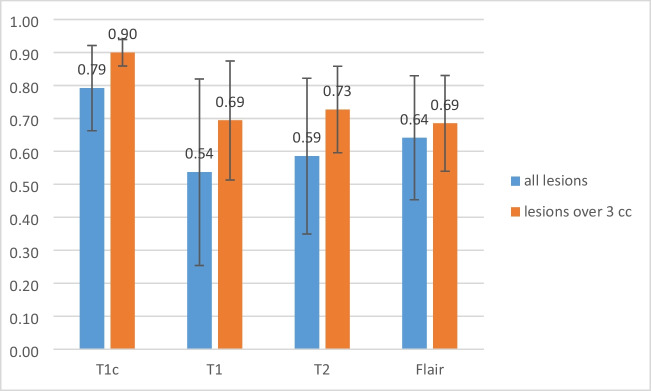
The average and standard deviation of DSC for inter-observer comparison for all lesions and lesions larger than 3 cm^3^, respectively

The average and standard deviation of DSC for inter-observer comparison were 0.79 ± 0.13, 0.54 ± 0.25, 0.59 ± 0.24, and 0.64 ± 0.19 for the pre-treatment T1c, online-T1, T2, and FLAIR respectively (blue bars in Fig. 2). The average and standard deviation of MDA for inter-observer comparison were 2.26 ± 2.13, 8.10 ± 9.02, 9.02 ± 9.66, and 5.61 ± 5.66 for the pre-treatment T1c, online-T1, T2, and FLAIR respectively. Inter-observer variations were significantly smaller for the pre-treatment T1c than for the contrast-free online MRI scans (DSC: *P* < 0.001, MDA: *P* < 0.05), while they were smaller in online-FLAIR than in the online-T1 and T2 images (DSC: *P* < 0.05). However, no significant difference was found between the contours of the online-T1 and T2 scans (*P* = 0.06).

For lesions larger than 3 cm^3^, the average and standard deviation of DSC for inter-observer comparison were 0.90 ± 0.04, 0.69 ± 0.18, 0.73 ± 0.13, and 0.69 ± 0.15 for GTVs generated on the pre-treatment T1c, online-T1, T2, and FLAIR respectively (orange bars in Fig. 2). The average and standard deviation of MDA for inter-observer comparison were 0.66 ± 0.34, 3.45 ± 3.87, 2.07 ± 1.47, and 2.33 ± 1.49 for the T1c, T1, T2, and FLAIR respectively. Inter-observer variations were significantly smaller for pre-treatment T1c compared to contrast-free online MRI scans (DSC: *P* < 0.001, MDA: *P* < 0.05). However, FLAIR did not show an advantage over T1 and T2 for online scans in terms of inter-observer variations.

### Intra-observer consistency

**Fig. 3:**
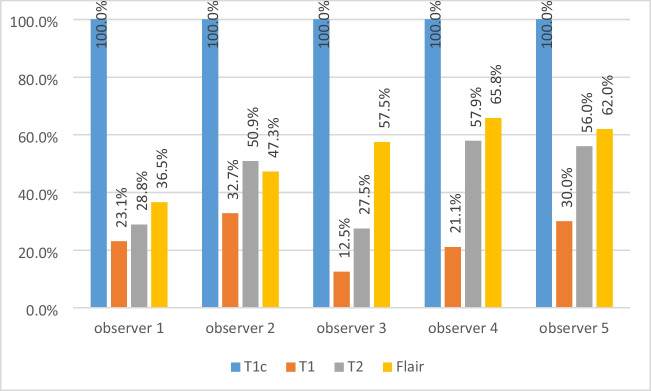
Visibility of lesions smaller than 3 cm^3^ for different online MR image sets (normalised to pre-treatment T1c images)

**Fig. 4:**
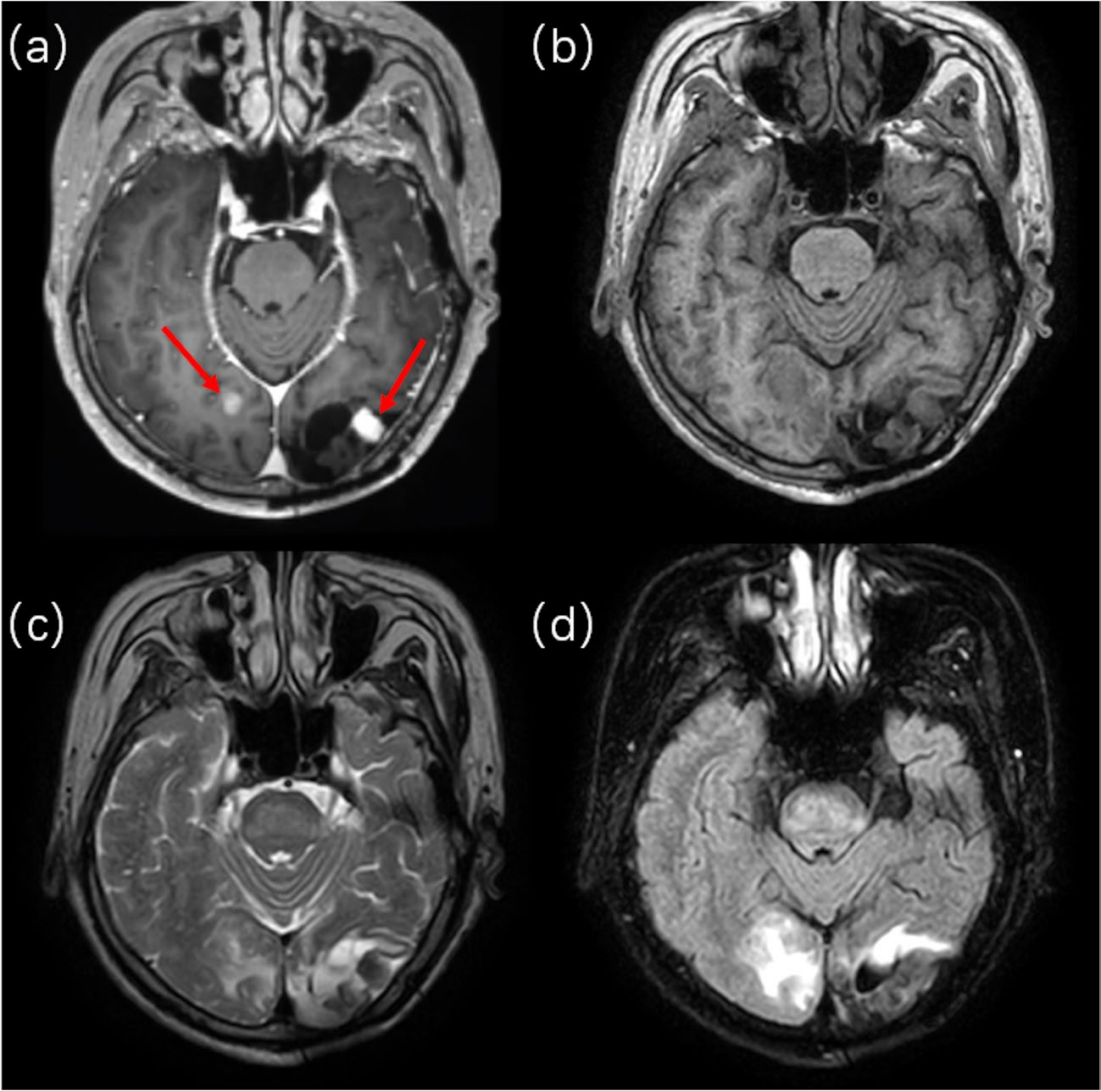
Two lesions were clearly visible on the right and left hemispheres, respectively, on (**a**) pre-treatment T1c image but difficult to locate in the images of (**b**) online-T1, (**c**) online-T2, and (**d**) online-FLAIR

Compared to the contrast-enhanced pre-treatment T1c image, the MR-Linac images showed significantly reduced visibility of the brain lesions, especially for lesions < 3 cm^3^ in size. Figure 3 shows the visibility of small lesions (< 3 cm^3^) in different online MRI scans compared to the pre-treatment T1c images for each observer. Figure 4 shows two lesions that were clearly visible on the T1c image but difficult to locate on the online MR-Linac images. These factors collectively led to poor online GTV conformity with the GTV-T1c in all patients.

**Fig. 5:**
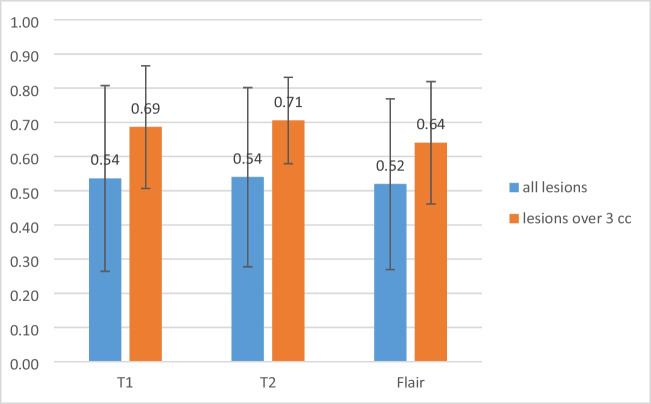
Average and standard deviation of DSC for intra-observer comparison between GTVs generated on online MRIs and pre-treatment T1c

Compared to the GTVs created on the pre-treatment T1c images, the average and standard deviation of DSC for intra-observer comparison were 0.54 ± 0.27, 0.54 ± 0.26, and 0.52 ± 0.25 for the online-T1, T2 and FLAIR scans respectively (blue bars in Fig. 5). The average and standard deviation of MDA for intra-observer comparison were 8.17 ± 11.67, 8.4 ± 11.72, and 8.37 ± 9.97 for the online-T1, T2, and FLAIR images respectively. No statistically significant differences were found between the different MR-Linac scans.

For lesions larger than 3 cm^3^, the average and standard deviation of DSC for intra-observer comparison were 0.69 ± 0.18, 0.71 ± 0.13, and 0.64 ± 0.18 for the online-T1, T2, and FLAIR respectively (orange bars in Fig. 5). The average and standard deviation of MDA for intra-observer comparison were 3.36 ± 7.00, 2.31 ± 3.37, and 2.86 ± 2.62 for online-T1, T2, and FLAIR respectively. T2 slightly outperformed FLAIR in terms of consistency with T1c images (*P* < 0.05). No significant difference was found between the online-T1 and T2 images for GTV contours.

### Performance of online contouring for adaptive treatment planning

**Fig. 6:**
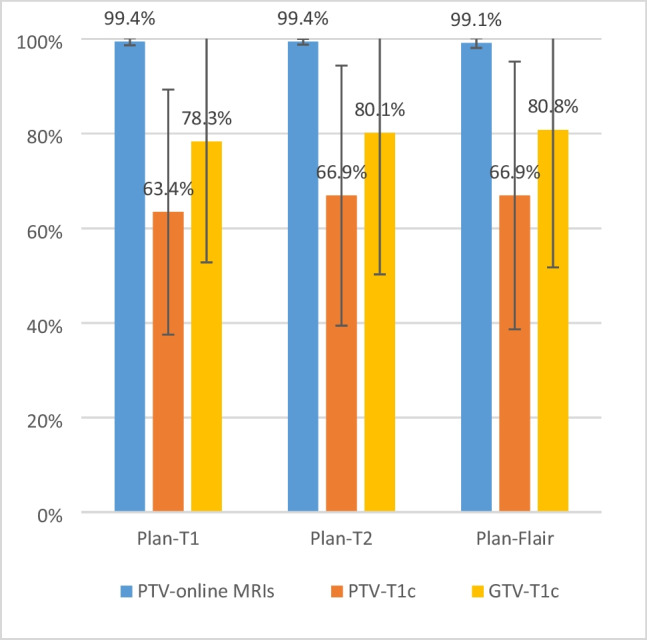
Prescription dose coverage (PDC) rate in simulated adaptive online planning evaluated with online contoured PTVs (blue) and “real” PTVs (orange) and GTVs (yellow) generated on pre-treatment T1c images, respectively

All PTVs generated with online MRI scans reached over 99% of the PDC rate in the simulated adaptive online planning. However, when evaluated using the GTVs and PTVs generated on contrast-enhanced T1w images (PTV-T1c), the PDC of the ground-truth targets significantly decreased. The average actual PDC rates of PTVs were 63.4%, 66.9%, and 66.9% for Plan-T1, Plan-T2, and Plan-FLAIR, respectively (Fig. 6). And the average actual PDC of GTVs were 78.3%, 80.1% and 80.8% for Plan-T1, Plan-T2, and Plan-FLAIR, respectively (Fig. 6).

When evaluated using the “real” targets generated on the pre-treatment T1c images, NCI in the simulated adaptive treatment planning increased significantly. The median NCI values for the PTVs generated on online MRI scans were 1.39, 1.36, and 1.29 for Plan T1, Plan T2, and Plan FLAIR, respectively. However, the values reached 1.9, 1.63, and 2.31, respectively, when evaluated using PTV-T1c.

## Discussions

In this study, we evaluated the accuracy of online target delineation for MRI-guided adaptive FSRT for BMs. Inter-observer analysis showed a mean DSC for GTV of 0.79, 0.54, 0.59 and 0.64 in the pre-treatment T1c, online T1, online-T2 ad online FLAIR images. Intra-observer analysis revealed low conformity between the GTVs contoured on online MRIs and pre-treatment T1c. The mean DSC with GTV-T1c merely reached 0.53 for GTVs contoured on non-enhanced online MRI images. Simulation of online adaptive planning showed that the average PDC rate fell from over 99% to 65.7% when evaluated using ground truth PTVs generated on contrast-enhanced T1w images. In summary, the accuracy of online target contouring needs to be improved for the current MRI-guided online adaptive FSRT.

This outcome aligned with our expectations, given that contrast-enhanced MRI is the standard diagnostic imaging modality for BMs. The injected GBCA can penetrate the diseased blood–brain barrier, causing tumour enhancement and making decisions regarding the tumour boundary less controversial among different observers. However, MR-Linac images without contrast injection showed significantly lower inter-observer DSCs, which could be attributed to both reduced image quality and the absence of contrast agents. Intra-observer analysis revealed low conformity between the GTVs contoured on online MRIs and pre-treatment T1c. Simulated adaptive planning showed significant drop in PDC of real targets. This would impair the confidence of online recontouring of tumour targets during the ART workflow and thus reduce the benefits of online ART. Notably, a PTV margin of 3 mm was applied in the simulated planning process, which reduced the PDC differences between the online GTVs and real targets (from 100% to 79.7%).

Compared to pre-treatment target contouring based on contrast-enhanced MRI and planning CT images, current online adaptive target contouring is challenging not only due to the limited time but also because of the lack of adequate tumour resolution in online images. Studies have shown that contouring results can differ significantly between observers for pre-treatment target delineation [23, 24]. Online ART could further increase the differences between the contours of different observers, as the absence of critical image modalities and reduced image resolution made it difficult to accurately distinguish lesions from normal tissues. A recent study found significant CTV contouring variability between diagnostic T1c and MR-Linac T2 FLAIR sequences for postoperative MRI of BMs [11]. However, no studies have investigated the accuracy of non-enhanced online MRI for GTV delineation.

The findings of this study suggest the need to improve the accuracy of online target delineation in MRgART. Considering that MRI techniques with different contrasts reveal different physiological information and are therefore not interchangeable, a combination of multi-contrast MRIs would be helpful for more accurate lesion delineation. However, the current acquisition of a single image set requires several minutes to complete, and an extension of the scanning time increases the likelihood of intra-fractional motion. Recent developments in multiparametric MRI, such as the introduction of MR fingerprinting technology, could help address this issue [25–27]. As MR fingerprinting technology made simultaneous acquisition of multi-contrast MRIs in single scan possible, significantly reducing time for image acquisitions.

However, for BM delineation, GBCA-enhanced MRI has a significant advantage in defining the tumour boundary; hence, it is unlikely to be replaced by other modalities in the near future [28, 29]. Thus, GBCA administration during MRI guided adaptive FSRT for patients with BMs should be considered [30, 31]. For patients with impaired renal function, a reduced GBCA dose or extended interval for contrast agent injections should be considered. However, former recommendations by the European Society of Urogenital Radiology and the American College of Radiology mainly focused on repeated injections of the contrast medium in a diagnostic scenario in which daily GBCA administration was not a common practice. Whereas, the application of MRgART requires daily GBCA administration, which might result in gadolinium retention inside the human body. Studies have found gadolinium deposits in the brain and other tissues in patients with a history of multiple GBCA administrations; though no adverse effects have yet been reported [32, 33]. Although Wang et al. have demonstrated the stability of the physicochemical properties of GBCA in laboratory conditions under 1.5 T magnetic and 7 MV X-ray radiation fields of the MR-Linac [34], in vivo studies are essential to exclude unexpected metabolites in biological environments. Thus, extra care should be taken when performing daily pre-treatment GBCA injections.

For patients who are reluctant to receive multiple contrast injections, radiation oncologists should rely on non-enhanced online MRIs for GTV contouring. In such cases, large and small lesions may show different preferences for reference MRI sequences. For lesions > 3 cm^3^, T2 performed significantly better than FLAIR in terms of consistency with the GTV-T1c (*P* < 0.05). This may result from the fact that, although FLAIR could visualise some pathological changes, the signal of peritumoural tissue oedema overwhelmed the tumour signal and made accurate delineation of the tumour even more challenging (Fig. 5). Whereas, for small lesions (< 3 cm^3^), FLAIR showed the best visibility among the online MRI systems. In four of the five observers, FLAIR outperformed T2 for small lesion visibility, and for all five observers, T2 outperformed T1 in small lesion visibility. Therefore, for patients with multiple BMs, acquiring T2 and FLAIR images is recommended for online contouring. Additionally, methods such as contrast-enhanced MRI synthesis from non-enhanced MRIs provide a new route to address this issue [35–38].

It should be noted that in this study, online MRI performed inferiorly to pre-treatment T1c in target delineations, not only due to the absence of contrast enhancement but also due to the reduced image quality of the MR-linac compared to diagnostic MRI. Although both systems were manufactured by the same vendor, the MRI simulator was superior to the onboard MRI in terms of the B0 field strength, gradient coils, and radiofrequency coils. This led to a much better signal-to-noise ratio of the MR signals, producing higher-quality images. This study focused on evaluating the accuracy of online contouring using the MRgART. Therefore, diagnostic T1c was selected as the ground truth for target contouring. The performance of non-contrast-enhanced diagnostic MRIs has not been studied separately. However, further improvement in the image quality of the MR-Linac was constrained by hardware potential. Therefore, GBCA administration is a more practical method to improve the accuracy of online target delineation in BMs. Whereas, differences in contouring may still exist between 1.5 T and 3.0 T CE-MRIs. Therefore, the uncertainty of GTV contouring on 1.5 T CE-MRIs should be evaluated in future studies to determine specific PTV margins for MRI-guided FSRT of BMs.

## Conclusions

The performance of non-enhanced MR-Linac images in tracking targets in online ART for BMs was unsatisfactory, as the average DSC merely reached 0.53 for all the contours on MR-Linac images compared to the contrast-enhanced MRI used for reference planning. Inaccuracy in contouring resulted in a falloff of one-third of the PDC for real target volume (from 99 to 66%). Small BMs often lack visibility in online MR without contrast enhancement. Hence, future studies should consider the feasibility of contrast agent administration during online adaptive treatment courses.

## Data Availability

The research data are stored in the institutional repository of Sun Yat-sen University Cancer Center and shared with the corresponding author upon request.

## Abbreviations

ART: Adaptive radiotherapy
BMs: Brain metastases
FSRT: Fractionated stereotactic radiotherapy
GBCA: Gadolinium-based contrast agent
GTVs: Gross target volume
MRI: Magnetic resonance imaging
MDA: Mean distance to agreement
MRgART: MRI-guided adaptive radiotherapy
NCI: New conformity index
PTVs: Planning target volume
PDC: Prescription dose coverage
SRS: Stereotactic radiosurgery
SRT: Stereotactic radiotherapy
